# Association of Blood-Based Brain Injury Biomarker Concentrations With Outcomes After Pediatric Cardiac Arrest

**DOI:** 10.1001/jamanetworkopen.2022.30518

**Published:** 2022-09-08

**Authors:** Ericka L. Fink, Patrick M. Kochanek, Ashok Panigrahy, Sue R. Beers, Rachel P. Berger, Hülya Bayir, Jose Pineda, Christopher Newth, Alexis A. Topjian, Craig A. Press, Aline B. Maddux, Frederick Willyerd, Elizabeth A. Hunt, Ashley Siems, Melissa G. Chung, Lincoln Smith, Jesse Wenger, Lesley Doughty, J. Wesley Diddle, Jason Patregnani, Juan Piantino, Karen Hallermeier Walson, Binod Balakrishnan, Michael T. Meyer, Stuart Friess, David Maloney, Pamela Rubin, Tamara L. Haller, Amery Treble-Barna, Chunyan Wang, Robert R. S. B. Clark, Anthony Fabio

**Affiliations:** 1 Department of Critical Care Medicine, UPMC Children’s Hospital of Pittsburgh, Pittsburgh, Pennsylvania; 2 Department of Pediatrics, UPMC Children’s Hospital of Pittsburgh, Pittsburgh, Pennsylvania; 3 Safar Center for Resuscitation Research, University of Pittsburgh Medical Center, Pittsburgh, Pennsylvania; 4 Department of Radiology, UPMC Children’s Hospital of Pittsburgh, Pittsburgh, Pennsylvania; 5 Department of Psychiatry, University of Pittsburgh School of Medicine, Pittsburgh, Pennsylvania; 6 Children’s Neuroscience Institute, UPMC Children’s Hospital of Pittsburgh, Pittsburgh, Pennsylvania; 7 Department of Anesthesiology Critical Care Medicine, Children’s Hospital Los Angeles, Los Angeles, California; 8 Department of Anesthesia and Critical Care Medicine, Children’s Hospital of Philadelphia, University of Pennsylvania School of Medicine, Philadelphia; 9 Department of Pediatrics and Neurology, Children’s Hospital of Philadelphia, University of Pennsylvania School of Medicine, Philadelphia; 10 Department of Pediatrics, Children’s Hospital Colorado, University of Colorado School of Medicine, Aurora; 11 Department of Pediatrics, Phoenix Children’s Hospital, Phoenix, Arizona; 12 Department of Anesthesiology and Critical Care Medicine, Johns Hopkins Children’s Center, Baltimore, Maryland; 13 Department of Pediatrics, Johns Hopkins Children’s Center, Baltimore, Maryland; 14 Department of Pediatrics, Divisions of Pediatric Neurology and Critical Care Medicine, Nationwide Children’s Hospital, Columbus, Ohio; 15 Department of Pediatrics, University of Washington School of Medicine, Seattle; 16 Department of Pediatrics, Cincinnati Children’s Hospital Medical Center, Cincinnati, Ohio; 17 Department of Pediatrics, Children’s National Hospital, District of Columbia; 18 Department of Pediatrics, Barbara Bush Children’s Hospital, Portland, Maine; 19 Department of Pediatrics, Oregon Health & Science University, Portland; 20 Department of Pediatrics, Children’s Healthcare of Atlanta, Atlanta, Georgia; 21 Department of Pediatrics, Children’s Wisconsin, Medical College of Wisconsin, Milwaukee; 22 Department of Pediatrics, St Louis Children’s Hospital, St Louis, Missouri; 23 Department of Epidemiology, University of Pittsburgh School of Medicine, Pittsburgh, Pennsylvania; 24 Department of Physical Medicine and Rehabilitation, University of Pittsburgh School of Medicine, Pittsburgh, Pennsylvania

## Abstract

**Importance:**

Families and clinicians have limited validated tools available to assist in estimating long-term outcomes early after pediatric cardiac arrest. Blood-based brain-specific biomarkers may be helpful tools to aid in outcome assessment.

**Objective:**

To analyze the association of blood-based brain injury biomarker concentrations with outcomes 1 year after pediatric cardiac arrest.

**Design, Setting, and Participants:**

The Personalizing Outcomes After Child Cardiac Arrest multicenter prospective cohort study was conducted in pediatric intensive care units at 14 academic referral centers in the US between May 16, 2017, and August 19, 2020, with the primary investigators blinded to 1-year outcomes. The study included 120 children aged 48 hours to 17 years who were resuscitated after cardiac arrest, had pre–cardiac arrest Pediatric Cerebral Performance Category scores of 1 to 3 points, and were admitted to an intensive care unit after cardiac arrest.

**Exposure:**

Cardiac arrest.

**Main Outcomes and Measures:**

The primary outcome was an unfavorable outcome (death or survival with a Vineland Adaptive Behavior Scales, third edition, score of <70 points) at 1 year after cardiac arrest. Glial fibrillary acidic protein (GFAP), ubiquitin carboxyl-terminal esterase L1 (UCH-L1), neurofilament light (NfL), and tau concentrations were measured in blood samples from days 1 to 3 after cardiac arrest. Multivariate logistic regression and area under the receiver operating characteristic curve (AUROC) analyses were performed to examine the association of each biomarker with outcomes on days 1 to 3.

**Results:**

Among 120 children with primary outcome data available, the median (IQR) age was 1.0 (0-8.5) year; 71 children (59.2%) were male. A total of 5 children (4.2%) were Asian, 19 (15.8%) were Black, 81 (67.5%) were White, and 15 (12.5%) were of unknown race; among 110 children with data on ethnicity, 11 (10.0%) were Hispanic, and 99 (90.0%) were non-Hispanic. Overall, 70 children (58.3%) had a favorable outcome, and 50 children (41.7%) had an unfavorable outcome, including 43 deaths. On days 1 to 3 after cardiac arrest, concentrations of all 4 measured biomarkers were higher in children with an unfavorable vs a favorable outcome at 1 year. After covariate adjustment, NfL concentrations on day 1 (adjusted odds ratio [aOR], 5.91; 95% CI, 1.82-19.19), day 2 (aOR, 11.88; 95% CI, 3.82-36.92), and day 3 (aOR, 10.22; 95% CI, 3.14-33.33); UCH-L1 concentrations on day 2 (aOR, 11.27; 95% CI, 3.00-42.36) and day 3 (aOR, 7.56; 95% CI, 2.11-27.09); GFAP concentrations on day 2 (aOR, 2.31; 95% CI, 1.19-4.48) and day 3 (aOR, 2.19; 95% CI, 1.19-4.03); and tau concentrations on day 1 (aOR, 2.44; 95% CI, 1.14-5.25), day 2 (aOR, 2.28; 95% CI, 1.31-3.97), and day 3 (aOR, 2.04; 95% CI, 1.16-3.57) were associated with an unfavorable outcome. The AUROC models were significantly higher with vs without the addition of NfL on day 2 (AUROC, 0.932 [95% CI, 0.877-0.987] vs 0.871 [95% CI, 0.793-0.949]; *P* = .02) and day 3 (AUROC, 0.921 [95% CI, 0.857-0.986] vs 0.870 [95% CI, 0.786-0.953]; *P* = .03).

**Conclusions and Relevance:**

In this cohort study, blood-based brain injury biomarkers, especially NfL, were associated with an unfavorable outcome at 1 year after pediatric cardiac arrest. Additional evaluation of the accuracy of the association between biomarkers and neurodevelopmental outcomes beyond 1 year is needed.

## Key Points

**Question:**

Are early blood-based brain injury biomarkers associated with an unfavorable outcome 1 year after pediatric cardiac arrest?

**Findings:**

In this cohort study of 120 children who were resuscitated after cardiac arrest, blood concentrations of 4 brain injury biomarkers (glial fibrillary acidic protein, ubiquitin carboxyl-terminal esterase L1, neurofilament light, and tau) were associated with unfavorable outcomes at 1 year.

**Meaning:**

These findings suggest that blood-based brain injury biomarkers for clinical use may aid in early outcome assessment after pediatric cardiac arrest.

## Teaser

This cohort study assesses the association between blood-based brain injury biomarker concentrations and 1-year outcomes among children who were resuscitated after cardiac arrest.

## Introduction

Approximately 10 000 children experience in-hospital or out-of-hospital cardiac arrest annually in the US.^1,2^ Children with return of spontaneous circulation (ROSC) are at high risk of neurological morbidity and death due to global hypoxic-ischemic brain injury.^3^ Accurate early understanding of the risk of neurological injury could support clinician and family decision-making and treatment. However, a standardized validated approach is lacking.^4^

Small observational studies have found that blood-based brain injury biomarkers may be associated with outcomes after pediatric cardiac arrest.^5,6^ The brain-specific biomarkers ubiquitin carboxyl-terminal esterase L1 (UCH-L1) and glial fibrillary acidic protein (GFAP) have been approved by the US Food and Drug Administration to assist in clinical decision-making for mild traumatic brain injury in adults and differentiated outcomes in a pilot study of pediatric cardiac arrest.^7,8,9^ The UCH-L1 enzyme, which is located in neurons, normally participates in the degradation of damaged proteins,^10^ and GFAP forms intermediate filaments that support the shape and function of astroglia cells.^11^ Furthermore, biomarkers of blood-based white matter injury are helpful tools after pediatric and adult cardiac arrest.^12,13^ Thus, neurofilament light (NfL), a component of the neuronal cytoskeleton providing structural support to axons,^14,15,16^ and tau, a microtubule-stabilizing neuroaxial protein that maintains stability of axon microtubules, were also included in this study.^17,18^

We conducted a prospective multicenter cohort study (Personalizing Outcomes After Child Cardiac Arrest [POCCA]) to evaluate the association of these 4 brain-specific biomarkers on days 1 to 3 after pediatric cardiac arrest with the composite outcome of death or unfavorable adaptive behavior at 1 year. We hypothesized that each blood-based biomarker would be associated with outcomes after cardiac arrest.

## Methods

### Study Design and Setting

The POCCA prospective cohort study was conducted in pediatric intensive care units (ICUs) at 14 academic referral centers in the US between May 16, 2017, and August 19, 2020, with follow-up through 1 year after enrollment. The institutional review board of the University of Pittsburgh served as the study’s central review board and approved the study for performance at UPMC Children’s Hospital of Pittsburgh. Two sites, Children’s Healthcare of Atlanta and Children’s Wisconsin, obtained independent institutional review board approval because they were unable to participate in the central review board. Written informed consent from a parent or guardian was required for participation, and written patient assent was obtained when appropriate (based on local center guidelines). This study followed the Strengthening the Reporting of Observational Studies in Epidemiology (STROBE) reporting guideline for cohort studies.^19^

### Participants

Eligible children were aged 48 hours to 17 years, had chest compressions performed for any duration of time for in-hospital or out-of-hospital events, and had a pre–cardiac arrest score of 1 to 3 points on the Pediatric Cerebral Performance Category scale (score range, 1-6 points, with 1 indicating good, 2 indicating mild disability, 3 indicating moderate disability, 4 indicating severe disability, 5 indicating vegetative state, and 6 indicating death).^20^ Children were excluded if they had a do-not-resuscitate order or were actively undergoing brain death evaluation at the time of screening, were in foster care or the judicial system, were pregnant, or did not have a blood sample (200 μL) available within 24 hours of cardiac arrest. Screening occurred daily. Patients did not receive any study-related treatment interventions. Clinical care was provided by the patient’s clinical team.

**Figure 1.**
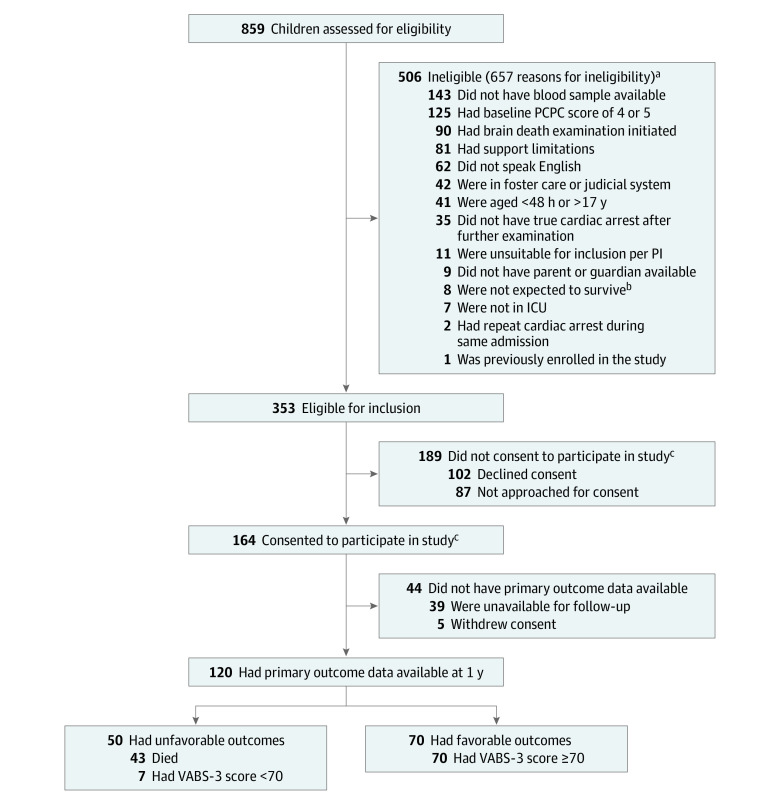
Study Flowchart ICU indicates intensive care unit; PCPC, Pediatric Cerebral Performance Category; PI, primary investigator; and VABS-3, Vineland Adaptive Behavior Scales, third edition. ^a^Children may have met 1 or more criteria that made them ineligible for the study. ^b^Criteria regarding likelihood of survival were determined by site research personnel; therefore, it was inappropriate to approach these patients for enrollment. ^c^Informed consent from a parent or guardian was required for participation, and patient assent was obtained when appropriate.

Among 932 children who experienced a cardiac arrest, 859 were assessed for eligibility. Children could have met 1 or more criteria that made them ineligible for participation. A total of 506 children were ineligible, with the most frequent criteria being lack of a blood sample within the first 24 hours after ROSC (143 patients [28.3%]), a pre–cardiac arrest Pediatric Cerebral Performance Category score of 4 points (indicating severe neurological disability) and/or 5 points (indicating coma; 125 patients [24.7%]), initiation of brain death evaluation (90 patients [17.8%]), and/or care limitations (81 patients [16.0%]). Among the remaining 353 children eligible for participation, 102 families (28.9%) declined consent, 87 families (24.6%) were not approached for consent, and 164 families (46.5%) provided informed consent; of those who provided consent, 44 children (26.8%) did not have primary outcome data available (39 children were unavailable for follow-up, and 5 families withdrew consent; 1 of the families who withdrew consent requested the child’s data be removed, resulting in 43 of 163 children [26.4%] without primary outcome data available). A total of 120 children with primary outcome data were included in the final study sample (Figure 1).

### Data Collection

Data were collected locally using a case report form. Cardiac arrest, resuscitation, and post-ROSC variables were collected from the medical record using Utstein definitions.^21,22^ Patient demographic characteristics were extracted from the medical record using categories recommended by the National Institutes of Health.^23^ Organ support, testing results, and some outcomes were collected from the medical record. Cause of death was abstracted from the death certificate. The score on the Pediatric Index of Mortality 3 (score range, 0-100, with higher scores indicating higher risk of death) was recorded using the first value of each variable measured from the ICU admission to 1 hour after arrival in the ICU or, for cardiac arrests occurring in the ICU, the score was recorded using the first value within 1 hour after ROSC.^24^

#### Blood-Based Biomarkers

Three milliliters of blood was collected prospectively on days 1 to 3 after ROSC. Day 1 was defined as the first 24 hours after ROSC, day 2 as 24 to 48 hours after ROSC, and day 3 as 48 to 72 hours after ROSC. Blood samples were centrifuged, aliquoted, frozen at −70 °C, and mailed to the University of Pittsburgh for storage and batched analysis. If prospective samples were not available, leftover serum or plasma could be obtained from the hospital’s laboratory and stored and mailed using the same procedure. Concentrations of NfL, UCH-L1, GFAP, and tau in serum or plasma were measured by a laboratory technician at an independent laboratory (Quanterix) using an assay specific to the 4 biomarkers of interest (Simoa Human Neurology 4-Plex A; Quanterix); the technician was blinded to patient data. Lower limits of quantitation from the assay were 0.241 pg/mL for NfL, 5.450 pg/mL for UCH-L1, 0.467 pg/mL for GFAP, and 0.053 pg/mL for tau. Samples were measured in duplicate; mean concentrations were used for analysis. Dilution factors of 4, 10, or 1000 were used. Clinical team members were blinded to the biomarker results. One child’s family withdrew all data, and another child did not have a blood sample available on day 1, leaving 162 samples for analysis. On days 2 and 3, samples were available from 141 patients and 128 patients, respectively.

### Outcome Measures

Patient follow-up was performed by each center. Parents or guardians completed the Vineland Adaptive Behavior Scales, third edition (VABS-3), rating form at 1 year via in-person, mail, or telephone follow-up.^25^ The VABS-3 is a standardized measure of adaptive behavior function based on caregiver report. The VABS-3 provides age-corrected standard scores (mean [SD], 100 [15] points) for individuals from birth through age 90 years in 4 domains (communication, daily living, socialization, and motor skills) and an overall adaptive behavior composite score, with higher scores denoting better functioning. A favorable outcome was defined as survival with a VABS-3 overall adaptive behavior composite score of 70 points or greater, and an unfavorable outcome was defined as a VABS-3 overall adaptive behavior composite score lower than 70 points or death, consistent with previous studies of children with cardiac arrest.^26,27^ Questionnaire responses were evaluated for quality and reliability by the primary study team.

### Statistical Analysis

A sample size of 164 patients achieved 80% power to detect an *R*^2^ of 0.10 for 15 independent variables (various combinations of trajectory groups with varying numbers of trajectories) using an *F* test with a significance threshold of α = .05. The variables tested were adjusted for an additional 2 independent variables, with an *R*^2^ of 0.10.^9^

Frequencies and percentages were reported for categorical variables. Biomarker data were log transformed and presented as medians with IQRs owing to nonparametric distributions. All patient and cardiac arrest variables were first evaluated as univariates for inclusion in the multivariate analysis^28^ using χ^2^ or Kruskal-Wallis tests as appropriate to examine the associations of characteristics and clinical features with 1-year outcomes. Covariates considered in the multivariate models included age, sex, race, ethnicity, preexisting conditions, cardiac arrest etiology, cardiac arrest location, duration of cardiopulmonary resuscitation, epinephrine boluses and defibrillations, first monitored rhythm, witnessed event status (a cardiac arrest that was seen or heard by another person or a cardiac arrest that was monitored^22^), bystander resuscitation (nonhospital personnel or hospital personnel not part of the emergency response system^22^), extracorporeal membrane oxygenation use, first blood pH and lactate levels, Pediatric Index of Mortality 3 score, first ICU Glasgow Coma Scale overall score (score range, 3-15 points, with higher scores indicating higher level of consciousness) and subscale scores (eye opening, verbal response, and motor response), and targeted temperature management (TTM) for fever prevention or therapeutic hypothermia. Covariates that were significant at *P* ≤ .20 were then evaluated using multivariable logistic regression models with stepwise selection and entry and removal levels of 0.20. We forced the inclusion of the log of the biomarker concentrations. Models were performed separately for each biomarker.

A multivariable analysis of the area under the receiver operating characteristic curve (AUROC) was used to assess the accuracy of each biomarker at each day by comparing the final logistic regression models with and without each biomarker among those who had unfavorable outcomes at 1 year. The threshold for statistical significance was 2-sided *P* < .05. Biomarker specificity, threshold (in picograms per milliliter), and sensitivity on univariate biomarker models were measured with a set specificity of 95% to minimize the chance of false assignment of an unfavorable outcome.

Missing data were not imputed. Only patients with primary outcome data available were analyzed. All analyses were conducted using SAS software, version 9.2 (SAS Institute, Inc).

## Results

### Participants

Of 120 children with primary outcome data available at 1 year, the median (IQR) age was 1.0 (0-8.5) year; 49 children (40.8%) were female, and 71 (59.2%) were male. A total of 5 children (4.2%) were Asian, 19 (15.8%) were Black, 81 (67.5%) were White, and 15 (12.5%) were of unknown race; of 110 children with data on ethnicity, 11 (10.9%) were Hispanic, and 99 (90.0%) were non-Hispanic. Patient characteristics, including race and ethnicity, were not different by outcome (Table 1). Compared with children who had a favorable outcome, those with an unfavorable outcome more frequently had out-of-hospital cardiac arrest (35 children [70.0%] vs 25 children [35.7%]), longer duration of cardiopulmonary resuscitation (median [IQR], 20.0 [6.0-40.0] minutes vs 5.0 [2.0-11.0] minutes), and an unwitnessed event (23 children [46.0%] vs 5 children [7.1%]) (Table 2).

**Table 1.** Patient Characteristics Overall and by Outcome Group

Characteristic	Patients, No./total No. (%)			*P* value^c^
	Overall (N = 120)	Favorable outcome (n = 70)^a^	Unfavorable outcome (n = 50)^b^	
Age, median (IQR), y	1.0 (0-8.5)	1.0 (0-9.0)	1.0 (0-6.0)	.55
Sex				
Female	49/120 (40.8)	28/70 (40.0)	21/50 (42.0)	.83
Male	71/120 (59.2)	42/70 (60.0)	29/50 (58.0)	
Race				
Asian	5/120 (4.2)	2/70 (2.9)	3/50 (6.0)	.71
Black	19/120 (15.8)	12/70 (17.1)	7/50 (14.0)	
White	81/120 (67.5)	46/70 (65.7)	35/50 (70.0)	
Unknown	15/120 (12.5)	10/70 (14.3)	5/50 (10.0)	
Ethnicity				
Hispanic	11/110 (10.0)	6/63 (9.5)	5/47 (10.6)	.85
Non-Hispanic	99/110 (90.0)	57/63 (90.5)	42/47 (89.4)	
Preexisting conditions^d^	77/113 (68.1)	44/63 (69.8)	33/50 (66.0)	.66
Cardiovascular	45/119 (37.8)	27/69 (39.1)	18/50 (36.0)	.73
Congenital	14/119 (11.8)	8/69 (11.6)	6/50 (12.0)	.95
Premature birth	24/110 (21.8)	18/62 (29.0)	6/48 (12.5)	.04
Neurological	11/120 (9.2)	5/70 (7.1)	6/50 (12.0)	.36
Pulmonary	25/120 (20.8)	14/70 (20.0)	11/50 (22.0)	.79
Cancer	3/120 (2.5)	2/70 (2.9)	1/50 (2.0)	.77
Organ or cell transplant	3/119 (2.5)	1/69 (1.4)	2/50 (4.0)	.38
Other	11/119 (9.2)	3/69 (4.3)	8/50 (16.0)	.03

^a^ A favorable outcome was defined as a Vineland Adaptive Behavior Scales, third edition (VABS-3), score of ≥70 points.

^b^ An unfavorable outcome was defined as a VABS-3 score of <70 points or death.

^c^ *P* values are based on a χ^2^ test for categorical variables and a Kruskal-Wallis test for continuous variables.

^d^ Patients may have had more than 1 preexisting condition.

**Table 2.** Cardiac Arrest, Resuscitation, Post–Cardiac Arrest, and Overall Outcomes at 1 Year After Cardiac Arrest by Outcome Group

Variable	Patients, No./total No. (%)			*P* value^c^
	Overall (N = 120)	Favorable outcome (n = 70)^a^	Unfavorable outcome (n = 50)^b^	
Primary etiology				
Asphyxia	74/107 (69.2)	45/66 (68.2)	29/41 (70.7)	.78
Cardiac	33/107 (30.8)	21/66 (31.8)	12/41 (29.3)	
Location out of hospital	60/120 (50.0)	25/70 (35.7)	35/50 (70.0)	<.001
Duration of cardiopulmonary resuscitation, median (IQR), min^d^	7.0 (3.0-20.0)	5.0 (2.0-11.0)	20.0 (6.0-40.0)	<.001
Epinephrine doses, median (IQR)^e^	1.0 (0-4.0)	1.0 (0-3.0)	2.5 (1.0-5.0)	.04
Received defibrillation	18/101 (17.8)	11/61 (18.0)	7/40 (17.5)	.94
First monitored rhythm				
Sinus bradycardia	33/96 (34.4)	23/58 (39.7)	10/38 (26.3)	.04
Pulseless electrical activity	23/96 (24.0)	14/58 (24.1)	9/38 (23.7)	
Asystole	19/96 (19.8)	7/58 (12.1)	12/38 (31.6)	
Ventricular tachycardia or fibrillation	15/96 (15.6)	10/58 (17.2)	5/38 (13.2)	
Other^f^	6/96 (6.3)	4/58 (6.9)	2/38 (5.3)	
Event witnessed	92/120 (76.7)	65/70 (92.9)	27/50 (54.0)	<.001
Bystander resuscitation	42/120 (35.0)	17/70 (24.3)	25/50 (50.0)	.004
Hospital length of stay, median (IQR), d	18.0 (6.5-35.5)	20.5 (10.0-41.0)	12.0 (5.0-34.0)	.09
ICU length of stay, median (IQR), d^g^	12.0 (5.0-25.0)	14.0 (6.0-21.0)	11.0 (5.0-31.5)	.78
Disposition at hospital discharge				
Home	55/120 (45.8)	50/70 (71.4)	5/50 (10.0)	<.001
Died	41/120 (34.2)	0	41/50 (82.0)	
Inpatient rehabilitation	17/120 (14.2)	15/70 (21.4)	2/50 (4.0)	
Transfer to other hospital	2/120 (1.7)	2/70 (2.9)	0	
Long-term care facility	5/120 (4.2)	3/70 (4.3)	2/50 (4.0)	
Days from cardiac arrest to death, median (IQR)^h^	10 (3-25)	NA	10 (3-25)	NA
Cause of death^i^				
Multiple organ failure	13/43 (30.2)	NA	13/43 (30.2)	NA
Brain death	11/43 (25.6)	NA	11/43 (25.6)	
Neurologic injury	11/43 (25.6)	NA	11/43 (25.6)	
Cardiovascular	8/43 (18.6)	NA	8/43 (18.6)	

Abbreviations: ICU, intensive care unit; NA, not applicable.

^a^ A favorable outcome was defined as a Vineland Adaptive Behavior Scales, third edition (VABS-3), score of ≥70 points.

^b^ An unfavorable outcome was defined as a VABS-3 score of <70 points or death.

^c^ *P* values are based on a χ^2^ test for categorical variables and a Kruskal-Wallis test for continuous variables.

^d^ Among 98 patients (64 with favorable outcomes and 34 with unfavorable outcomes).

^e^ Among 100 patients (60 with favorable outcomes and 40 with unfavorable outcomes).

^f^ Normal sinus, sinus tachycardia, and junctional ectopic tachycardia.

^g^ Among 117 patients (69 with favorable outcomes and 48 with unfavorable outcomes).

^h^ Among 43 patients (43 with unfavorable outcomes).

^i^ Per death certificate.

Among all 120 children, 79 children (65.8%) survived. Of the 43 children who died, 2 died after hospital discharge and before 1 year. Of those who survived, 70 children (88.6%) had a favorable outcome, and 9 (11.4%) had an unfavorable outcome. Thus, overall, 70 children (58.3%) had a favorable outcome, and 50 (41.7%) had an unfavorable outcome.

### Postresuscitation Data and Outcomes

Almost all children (117 patients [97.5%]) received mechanical ventilation, and vasoactive infusions were frequently used after ROSC (158 total infusions, with most children receiving epinephrine [76 infusions]) (eTable 1 in Supplement 1). Extracorporeal membrane oxygenation was used in 28 children (23.3%), with extracorporeal cardiopulmonary resuscitation representing most indications (22 children [78.6%]). Targeted temperature management to prevent fever was used more frequently in children with unfavorable vs favorable outcomes (18 children [36.0%] vs 12 children [17.1%]; *P* = .03), whereas TTM for therapeutic hypothermia was used in 12 children (17.1%) with a favorable outcome vs 3 children (6.0%; *P* = .07) with an unfavorable outcome.

### Blood Biomarker Concentrations by Outcome Group

**Figure 2.**
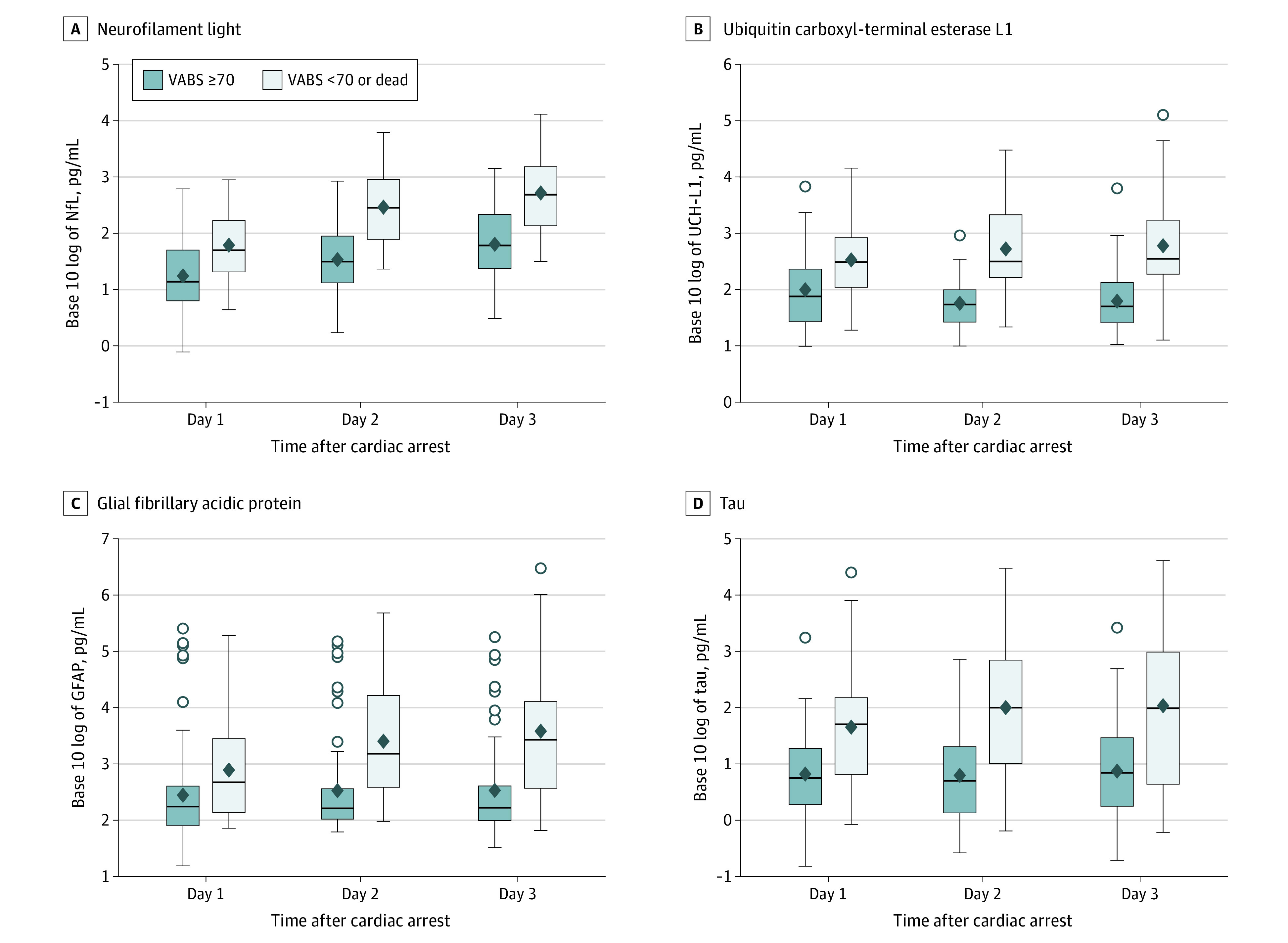
Log-Transformed Biomarker Concentrations Overall and by Outcome Group The Vineland Adaptive Behavior Scales, third edition (VABS-3) provides age-corrected standard scores (mean [SD], 100 [15] points) for individuals from birth through age 90 years in 4 domains (communication, daily living, socialization, and motor skills) and an overall adaptive behavior composite score, with higher scores denoting better functioning. A favorable outcome was defined as a VABS-3 overall adaptive behavior composite score of ≥70 points, and an unfavorable outcome was defined as a VABS-3 overall adaptive behavior composite score of <70 points or death. *P* < .001 for each comparison (with the exception of GFAP on day 1 [*P* = .002]) using a Kruskal-Wallis test. Circles represent outlier data points, diamonds represent statistically significant associations, and whiskers represent 95% CIs. GFAP indicates glial fibrillary acidic protein; NfL, neurofilament light; UCH-L1, ubiquitin carboxyl-terminal esterase L1.

Biomarker concentrations by outcome group on days 1, 2, and 3 after cardiac arrest are shown as picogram per milliliter concentrations in eTable 2 in Supplement 1 and as log-transformed concentrations in Figure 2. Biomarker concentrations were higher in children with unfavorable vs favorable 1-year outcomes for each of the 4 biomarkers studied. For example, on day 1, median (IQR) concentrations in the unfavorable vs favorable outcome groups were 50.54 (20.56-169.00) pg/mL vs 13.81 (6.29-49.98) pg/mL (*P* < .001) for NfL, 310.40 (110.73-848.36) pg/mL vs 73.39 (25.36-230.77) pg/mL (*P* < .001) for UCH-L1, 469.88 (137.70-2780.48) pg/mL vs 174.85 (79.63-401.27) pg/mL (*P* = .002) for GFAP, and 50.10 (6.53-149.30) pg/mL vs 5.59 (1.89-18.85) pg/mL (*P* < .001) for tau.

### AUROCs, Sensitivity, and Specificity of Biomarkers by Outcome Group

Univariate AUROCs for the outcome of individual biomarkers at days 1 to 3 are shown in eTable 3 in Supplement 1. On day 1, NfL had the best accuracy for 1-year outcome (AUROC, 0.731; 95% CI, 0.642-0.820), improving to 0.824 (95% CI, 0.742-0.907) on day 3. The best accuracy for 1-year outcome on day 2 (AUROC, 0.860; 95% CI, 0.785-0.935) and day 3 (AUROC, 0.837; 95% CI, 0.747-0.926) was observed for UCH-L1.

Biomarker sensitivity and threshold values selected to minimize false-positive identification of unfavorable outcomes for each day are shown in eTable 3 in Supplement 1. On all study days, 3 children were falsely classified as having an unfavorable outcome based on all 4 biomarkers, with the exception of day 2, during which UCH-L1 false-positive results occurred for 2 children. When purposely fixing specificity to 95%, tau had the best sensitivity of the 4 biomarkers on day 1 (AUROC, 0.333; 95% CI, 0.204-0.484), and UCH-L1 had the best sensitivity on day 2 (AUROC, 0.581; 95% CI, 0.421-0.730) and day 3 (AUROC, 0.605; 95% CI, 0.434-0.760).

### Multivariate Logistic Regression and AUROC Analyses of Unfavorable Outcome

For each day, multivariate logistic regression analyses were performed for individual biomarkers to assess their association with 1-year outcomes, with adjustment for patient and clinical covariates (Table 3; eTable 4 in Supplement 1). Concentrations of the following biomarkers were associated with unfavorable 1-year outcomes: NfL on post–cardiac arrest day 1 (adjusted odds ratio [aOR], 5.91; 95% CI, 1.82-19.19), day 2 (aOR, 11.88; 95% CI, 3.82-36.92), and day 3 (aOR, 10.22; 95% CI, 3.14-33.33; UCH-L1 on day 2 (aOR, 11.27; 95% CI, 3.00-42.36) and day 3 (aOR, 7.56 ; 95% CI, 2.11-27.09); GFAP on day 2 (aOR, 2.31; 95% CI, 1.19-4.48) and day 3 (aOR, 2.19; 95% CI, 1.19-4.03); and tau on day 1 (aOR, 2.44; 95% CI, 1.14-5.25), day 2 (aOR, 2.28; 95% CI, 1.31-3.97), and day 3 (aOR, 2.04; 95% CI, 1.16-3.57). The covariates most frequently associated with unfavorable outcomes across the multivariate models were unwitnessed events (eg, aORs for witnessed events on day 1: 0.15 [95% CI, 0.03-0.84] for NfL, 0.14 [95% CI, 0.03-0.81] for UCH-L1, 0.17 [95% CI, 0.03-0.91] for GFAP, and 0.11 [95% CI, 0.02-0.71] for tau), higher Pediatric Index of Mortality 3 score (eg, aORs on day 1: 2.47 [95% CI, 1.54-3.97] for NfL, 2.29 [95% CI, 1.44-3.64] for UCH-L1, 2.47 [95% CI, 1.56-3.90] for GFAP, and 2.10 [95% CI, 1.35-3.26] for tau), and lack of TTM for hypothermia (eg, aORs for use of TTM for therapeutic hypothermia on day 1: 0.02 [95% CI, 0-0.76] for NfL, 0.02 [95% CI, 0.001-0.37] for UCH-L1, 0.02 [95% CI, 0.001-0.38] for GFAP, and 0.02 [95% CI, 0.001-0.53] for tau) (Table 3). Multivariate AUROCs for outcomes with and without individual biomarkers for days 1 to 3 are shown in eTable 5 in Supplement 1. The models were significantly higher with vs without the addition of NfL for day 2 (AUROC, 0.932 [95% CI, 0.877-0.987] vs 0.871 [95% CI, 0.793-0.949]; *P* = .02) and day 3 (AUROC, 0.921 [95% CI, 0.857-0.986] vs 0.870 [95% CI, 0.786-0.953]; *P* = .03).

**Table 3.** Stepwise Multivariate Logistic Regression Analysis of 1-Year Favorable vs Unfavorable Outcomes on Days 1-3 After Cardiac Arrest by Biomarker^a^

Biomarker	aOR (95% CI)^b^		
	Day 1	Day 2	Day 3
**Neurofilament light**			
Concentration, log pg/mL	5.91 (1.82-19.19)	11.88 (3.82-36.92)	10.22 (3.14-33.33)
Age	0.90 (0.79-1.04)	NA	NA
Male vs female sex	0.35 (0.10-1.23)	0.67 (0.20-2.31)	NA
Cardiac vs asphyxia etiology	2.55 (0.70-9.34)	NA	1.96 (0.50-7.68)
Event witnessed	0.15 (0.03-0.84)	NA	NA
PIM-3 score	2.47 (1.54-3.97)	2.09 (1.39-3.14)	1.92 (1.29-2.86)
TTM used for therapeutic hypothermia	0.02 (0-0.76)	0.04 (0.002-0.70)	0.08 (0.004-1.55)
**Ubiquitin carboxyl-terminal esterase L1**			
Concentration, log pg/mL	2.01 (0.84-4.84)	11.27 (3.00-42.36)	7.56 (2.11-27.09)
Age	0.90 (0.79-1.01)	0.91 (0.79-1.04)	0.89 (0.76-1.05)
Male vs female sex	0.30 (0.09-1.01)	NA	0.56 (0.14-2.29)
Cardiac vs asphyxia etiology	2.61 (0.76-8.95)	NA	2.00 (0.47-8.54)
Event witnessed	0.14 (0.03-0.81)	0.09 (0.01-0.67)	0.11 (0.01-0.94)
PIM-3 score	2.29 (1.44-3.64)	1.44 (0.93-2.21)	1.56 (0.97-2.49)
TTM used for therapeutic hypothermia	0.02 (0.001-0.37)	0.02 (0.001-0.62)	0.02 (0.001-0.83)
**Glial fibrillary acidic protein**			
Concentration, log pg/mL	1.36 (0.72-2.59)	2.31 (1.19-4.48)	2.19 (1.19-4.03)
Age	0.88 (0.79-0.99)	0.90 (0.79-1.02)	0.92 (0.81-1.04)
Male vs female sex	0.30 (0.09-0.98)	0.30 (0.08-1.09)	0.48 (0.15-1.60)
Cardiac vs asphyxia etiology	3.31 (0.94-11.65)	3.82 (0.94-15.58)	NA
Event witnessed	0.17 (0.03-0.91)	0.15 (0.03-0.87)	0.13 (0.02-0.69)
PIM-3 score	2.47 (1.56-3.90)	1.96 (1.23-3.11)	1.71 (1.13-2.58)
TTM used for prevention of fever	NA	NA	1.48 (0.42-5.18)
TTM used for therapeutic hypothermia	0.02 (0.001-0.38)	2.31 (1.19-4.48)	0.05 (0.003-0.92)
**Tau**			
Concentration, log pg/mL	2.44 (1.14-5.25)	2.28 (1.31-3.97)	2.04 (1.16-3.57)
Age	0.90 (0.80-1.02)	0.93 (0.83-1.04)	0.97 (0.87-1.10)
Male vs female sex	0.24 (0.07-0.87)	0.53 (0.16-1.73)	0.58 (0.19-1.79)
Cardiac vs asphyxia etiology	2.25 (0.65-7.84)	NA	NA
Event witnessed	0.11 (0.02-0.71)	0.14 (0.03-0.68)	NA
PIM-3 score	2.10 (1.35-3.26)	1.79 (1.19-2.68)	1.87 (1.27-2.75)
TTM used for prevention of fever	NA	NA	1.90 (0.59-6.14)
TTM used for therapeutic hypothermia	0.02 (0.001-0.53)	0.06 (0.004-0.72)	0.16 (0.02-1.17)

Abbreviations: aOR, adjusted odds ratio; NA, not applicable (the variable did not meet criteria to stay in the model); PIM-3, Pediatric Index of Mortality 3; TTM, targeted temperature management.

^a^ Stepwise selection with entry and stay level of 0.20, forcing the inclusion of log biomarker concentration into models.

^b^ Wald 95% CIs.

## Discussion

To our knowledge, this cohort study is the largest analysis to date of the association between prospective blood-based brain injury biomarker concentrations and pediatric cardiac arrest. Results revealed that each of the 4 blood-based brain injury biomarkers that were analyzed early in the post–cardiac arrest period discriminated between favorable and unfavorable 1-year outcomes with high accuracy.^29^ These results remained significant after adjustment for common clinical factors associated with outcomes, including unwitnessed event status and risk of death score at admission.^30^

The NfL and UCH-L1 biomarkers both had high overall accuracy (based on the univariate AUROC analysis) and reliability (over the period examined) for 1-year outcomes. Only NfL had significant implications for multivariate AUROCs on days 2 and 3. Concentrations of NfL in children with both favorable and unfavorable outcomes numerically increased over the study days, potentially representing ongoing neuroaxonal injury.^31,32^ Our data are consistent with a single-center study^15^ involving children with cardiac arrest who experienced acute respiratory distress syndrome; that study found that NfL was associated with unfavorable outcomes at hospital discharge (as measured by Pediatric Cerebral Performance Category scores at discharge). A multicenter study^16^ that examined biobank samples from 717 adults with cardiac arrest who participated in a randomized clinical trial also found that NfL concentration was the best-performing biomarker compared with neuron-specific enolase, S100 calcium-binding protein B, and tau for the assessment of unfavorable outcomes based on the Cerebral Performance Category Scale at 6 months. Another study^33^ reported that the accuracy of NfL was better in adults (AUROC, 0.94-0.95 within the first 3 days after cardiac arrest) compared with the children in our study, which could reflect differences associated with patient developmental status, cardiac arrest phenotype, and/or patient selection. All patients in the adult clinical trial^33^ had an out-of-hospital cardiac arrest and presumed cardiac etiology and were comatose at the time of recruitment, whereas our study criteria were intentionally broad, with the aim of assessing biomarker performance across a heterogeneous cohort (eg, out-of-hospital and in-hospital cardiac arrest, asphyxia, and cardiac etiology) of children surviving to ICU admission (without known care limitations) to achieve greater generalizability and support biomarker translation into general clinical practice.

The UCH-L1 and GFAP biomarkers, both of which were approved by the Food and Drug Administration for use in clinical decision-making among those with mild traumatic brain injury, had the highest accuracy for 1-year outcomes on days 2 and 3 after cardiac arrest, replicating the findings reported in a pilot study.^7,8^ The UCH-L1 biomarker had the highest sensitivity of the 4 biomarkers when optimizing specificity on days 2 and 3. Notably, median UCH-L1 concentrations remained unchanged in both outcome groups over the 3 days, whereas GFAP concentrations increased in patients with unfavorable outcomes over the study period. A possible reason for this increase in GFAP levels is the fact that GFAP is both released and induced by injured and/or dying astrocytes.^23^ For some biomarkers, dynamic trajectories may offer more information than a single time point.^34^

Our study has several clinical implications. First, although most surviving children had a favorable 1-year outcome according to VABS-3 scores, they remained at risk of cognitive dysfunction.^35^ Children may not be neurologically assessable on examination early after resuscitation because of the need for sedative or neuromuscular blockade medications.^36^ The primary goal of the POCCA study was to provide clinicians and families with early and accurate tests to assist in clinical decision-making. Testing results can be used to facilitate discussion regarding planning for rehabilitative needs or determining goals of care and use of technological support.^9^ We found that blood-based brain injury biomarker testing, especially testing of NfL, was an accurate method to use when considering the odds of a child’s unfavorable composite outcome of death or unfavorable adaptive behavior function at 1 year in multivariate modeling. Our findings, as well as those of other groups,^15,16^ could support blood-based biomarker translation into pediatric clinical practice. In addition, these biomarkers could be used as a tool for estimation of enrichment in clinical trials if applied to identify patients who are most or least likely to benefit from a neuroprotective intervention. The biomarkers may also serve as surrogate outcomes to evaluate responsiveness to interventions.^37,38^ Future directions for research include testing blood-based brain injury biomarkers together and in combination with other clinical variables (eg, unwitnessed events, Pediatric Index of Mortality 3 score, and TTM) and tests (eg, brain imaging) to identify clinical phenotypes associated with outcomes that may be useful in future precision interventional clinical trials.^39,40,41^ In addition, assessment of the associations of early blood-based brain injury biomarkers with longitudinal neurodevelopmental and neuropsychological outcomes is needed.

### Limitations

This study has several limitations. Most children were ineligible for the study because they did not have a blood sample available within the first 24 hours after ROSC (28.3%), had a pre–cardiac arrest Pediatric Cerebral Performance Category score of 4 points (indicating severe neurological disability) and/or 5 points (indicating coma; 24.7%), were undergoing brain death evaluation (17.8%), and/or had care limitations (16.0%). A total of 28.9% of families in our study declined consent compared with those who declined consent in large out-of-hospital (27%) and in-hospital (55%) clinical trials.^26,27^ The patient sample size decreased over days 2 to 3 because of deaths and lack of blood samples. Biomarker concentrations were measured in the first 3 days; however, obtaining later blood samples, alone and together with other biomarkers and clinical variables, may improve accuracy. Our biomarker sampling strategy was pragmatic, acknowledging the need to prevent additional blood sample collection for safety (eg, surplus laboratory samples and risk of infection because of in-dwelling catheters). Biomarkers were measured by a private company using proprietary assays because clinical laboratory measurements are currently unavailable. Thus, comparisons of biomarker values between this study and others may be difficult to interpret.

We did not assess detailed neurodevelopmental outcomes and outcomes within the past year, both of which could potentially enhance the utility of blood-based brain injury biomarkers. Postresuscitation care, including the use of TTM, was not standardized in this study. Thus, findings associated with TTM and outcomes should be interpreted with caution. The outcome analysis was not adjusted for baseline function before cardiac arrest. However, children with baseline Pediatric Cerebral Performance Category scores of 4 or 5 points were excluded from the study, and the Pediatric Cerebral Performance Category scale performs similarly to the unfavorable VABS-3 threshold used in this study.^42^ One-year outcomes were unavailable for 43 children (26.4%) despite rigorous standard operating procedures and site training, with some notable differences in patient and hospital characteristics (eTable 6 in Supplement 1). Data regarding do-not-resuscitate status and deaths occurring after withdrawal of life-sustaining therapies were not collected in this study. The small sample in this study limited our ability to assess the consequences of coacute conditions such as sepsis; enrollment of patients with coacute severe brain injury was discouraged.

## Conclusions

This cohort study found that blood-based brain injury biomarkers, especially NfL at days 2 and 3 after cardiac arrest, were associated with the composite outcome of death or unfavorable adaptive behavior at 1 year after pediatric cardiac arrest with a high degree of accuracy. Evaluation of the accuracy of the association between these biomarkers and neurodevelopmental outcomes beyond 1 year is needed.

## Notes

Supplement 1. **eTable 1.** Postresuscitation Hospital Data Overall and by Favorable and Unfavorable Outcome Groups **eTable 2.** Unadjusted Biomarker Concentrations on Days 1 to 3 Overall, by Outcome Group, and by Alive With Unfavorable Outcome and Death at 1 Year After Cardiac Arrest **eTable 3.** Biomarker Area Under the Receiver Operating Characteristic Curves (AUROCs), Specificity, Threshold (pg/mL), and Sensitivity on Univariate Biomarker Models, With Set Specificity of 95% for Favorable vs Unfavorable Outcome **eTable 4.** Univariate Logistic Regression Models for Favorable or Unfavorable Outcome at 1 Year by Blood-Based Brain Injury Biomarker on Post–Cardiac Arrest Days 1, 2, and 3 **eTable 5.** Multivariate Area Under the Receiver Operating Characteristic Curves (AUROCs) With and Without Individual Biomarkers on Days 1, 2, and 3 **eTable 6.** Comparison of Patient and Cardiac Arrest Characteristics Among Children With and Without Data Available on 1 Year Outcomes

Supplement 2. **Nonauthor Collaborators.** Personalizing Outcomes After Child Cardiac Arrest (POCCA) Investigators
