# Supplementary material for: Association of Blood-Based Brain Injury Biomarker Concentrations With Outcomes After Pediatric Cardiac Arrest
Source: JAMA Netw Open. 2022 Sep 8;5(9):e2230518. doi: 10.1001/jamanetworkopen.2022.30518 (PMC9459665; doi:10.1001/jamanetworkopen.2022.30518)
Supplement: Supplement 1. — eTable 1. Postresuscitation Hospital Data Overall and by Favorable and Unfavorable Outcome Groups eTable 2. Unadjusted Biomarker Concentrations on Days 1 to 3 Overall, by Outcome Group, and by Alive With Unfavorable Outcome and Death at 1 Year After Cardiac Arrest eTable 3. Biomarker Area Under the Receiver Operating Characteristic Curves (AUROCs), Specificity, Threshold (pg/mL), and Sensitivity on Univariate Biomarker Models, With Set Specificity of 95% for Favorable vs Unfavorable Outcome eTable 4. Univariate Logistic Regression Models for Favorable or Unfavorable Outcome at 1 Year by Blood-Based Brain Injury Biomarker on Post–Cardiac Arrest Days 1, 2, and 3 eTable 5. Multivariate Area Under the Receiver Operating Characteristic Curves (AUROCs) With and Without Individual Biomarkers on Days 1, 2, and 3 eTable 6. Comparison of Patient and Cardiac Arrest Characteristics Among Children With and Without Data Available on 1 Year Outcomes [file jamanetwopen-e2230518-s001.pdf]

Supplementary Online Content

Fink EL, Kochanek PM, Panigrahy A, et al; Personalizing Outcomes After Child Cardiac Arrest (POCCA) Investigators. Association of blood-based brain injury biomarker concentrations with outcomes after pediatric cardiac arrest. *JAMA Netw Open*. 2022;5(9):e2230518. doi:10.1001/jamanetworkopen.2022.30518

eTable 1. Postresuscitation Hospital Data Overall and by Favorable and Unfavorable Outcome Groups

eTable 2. Unadjusted Biomarker Concentrations on Days 1 to 3 Overall, by Outcome Group, and by Alive With Unfavorable Outcome and Death at 1 Year After Cardiac Arrest

eTable 3. Biomarker Area Under the Receiver Operating Characteristic Curves (AUROCs), Specificity, Threshold (pg/mL), and Sensitivity on Univariate Biomarker Models, With Set Specificity of 95% for Favorable vs Unfavorable Outcome

eTable 4. Univariate Logistic Regression Models for Favorable or Unfavorable Outcome at 1 Year by Blood-Based Brain Injury Biomarker on Post–Cardiac Arrest Days 1, 2, and 3

eTable 5. Multivariate Area Under the Receiver Operating Characteristic Curves (AUROCs) With and Without Individual Biomarkers on Days 1, 2, and 3

eTable 6. Comparison of Patient and Cardiac Arrest Characteristics Among Children With and Without Data Available on 1 Year Outcomes

This supplementary material has been provided by the authors to give readers additional information about their work.

© 2022 Fink EL et al. *JAMA Network Open*.

eTable 1. Postresuscitation Hospital Data Overall and by Favorable and Unfavorable Outcome Groups

Variables, n (%)	Overall N=120	Favorable N=70	Unfavorable N=50	p-value
**Fever (> 38°C)**	59 (49.2)	34 (48.6)	25 (50.0)	0.877
**Seizure occurrence**	33 (27.5)	12 (17.1)	21 (42.0)	0.003
**Arterial blood gas values, median (interquartile range [IQR]), day 1**				
Lowest PaO2, mmHg	63.5 (48.0, 83.0) N=94	60.0 (46.5, 74.8) N=52	71.0 (55.0, 88.0) N=42	0.062
Highest PaO2, mmHg	193.5 (105.0, 283.7) N=94	235.5 (119.0, 325.0) N=52	169.8 (100.0, 239.0) N=42	0.048
Lowest PaCO2, mmHg	30.4 (27.0, 36.0) N=94	30.0 (27.7, 35.9) N=52	30.7 (26.0, 36.5]n=42	0.948
Highest PaCO2, mmHg	50.2 (42.5, 63.7) N=94	53.5 (43.5, 67.5) N=52	44.0 (38.7, 52.0) N=50	0.003
**Lowest systolic blood pressure, median (IQR), mmHg**	67.0 (55.5, 79.5) N=120	67.5 (58.0, 80.0) N=70	65.5 (52.0, 78.0) N=50	0.377
**Highest systolic blood pressure, median (IQR), mmHg**	124.0 (111.0, 141.5) N=120	122.0 (109.0, 140.0) N=70	125.5 (114.0, 144.0) N=50	0.583
**Invasive mechanical ventilation**	117 (97.5)	67 (95.7)	50 (100.0)	0.138
**Invasive mechanical ventilation, median (IQR), days**	8.0 (4.0, 16.0) N=117	8.0 (4.0, 13.0) N=67	9.5 (4.0, 22.0) N=50	0.278
**Vasoactive infusions, day 1**				
Epinephrine	76 (63.33)	76 (63.33)	76 (63.33)	0.370
Milrinone	28 (23.33)	17 (24.29)	11 (22.00)	0.770
Norepinephrine	22 (18.33)	10 (14.29)	12 (24.00)	0.175
Dopamine	13 (10.83)	5 (7.14)	8 (16.00)	0.124
Vasopressin	12 (10.00)	5 (7.14)	7 (14.00)	0.217
Phenylephrine	6 (5.00)	4 (5.71)	2 (4.00)	0.671
Isoproterenol	1 (0.83)	1 (1.43)	0 (0)	0.396
Dobutamine	0	0	0	NA

© 2022 Fink EL et al. *JAMA Network Open*.

**First lactate, median (IQR), mmol/L**	5.8 (2.9, 10.9) N=107	4.8 (2.3, 8.9) N=62	8.6 (3.8, 13.2) N=45	0.014
**Pediatric Index of Mortality, median (IQR), %**	16 (7-31)	14 (5-18)	29 (15-88)	<0.001
**First Glasgow Coma Scale score in the ICU, median (IQR)**	4 (3, 9) N=88	7 (3, 11) N=48	3 (3, 7) N=40	0.016
**Extracorporeal membrane oxygenation**	28 (23.3)	18 (25.7)	10 (20.0)	0.466
Extracorporeal CPR	22 (78.6)	15 (83.3)	7 (70.0)	0.410
**Target temperature management, prevention of fever**	31 (25.8)	12 (17.1)	18 (36.0)	0.032
**Target temperature management, therapeutic hypothermia**	15 (12.5)	12 (17.1)	3 (6.0)	0.069
Duration at target temperature, median (IQR), h	59 (40, 82)	59 (41, 82)	62 (19, 130)	0.942

ICU, intensive care unit; CPR, cardiopulmonary resuscitation. p-value are based on Chi-square test for categorical variables, Kruskal-Wallis test for continuous variables. Favorable outcome was Vineland Adaptive Behavioral Scale-III (VABS) score >=70 and Unfavorable outcome was VABS < 70 or died.

© 2022 Fink EL et al. *JAMA Network Open*.

eTable 2. Unadjusted Biomarker Concentrations on Days 1 to 3 Overall, by Outcome Group, and by Alive With Unfavorable Outcome and Death at 1 Year After Cardiac Arrest

A. Day 1

Median (interquartile range)	**Overall N=120**	**Favorable N=70**	**Unfavorable N=50**	**Kruskal-Wallis p-value**	**VABS < 70 N=7**	**Dead N=43**
**NFL**	n=118	n=70	n=48	<0.001	n=7	n=41
	27.95 (9.88, 70.75)	13.81 (6.29, 49.98)	50.54 (20.56, 169.00)		14.17 (9.88, 41.39)	62.40 (28.70, 199.00)
**Tau**	n=118	n=70	n=48	<0.001	n=7	n=41
	8.97 (2.85, 60.70)	5.59 (1.89, 18.85)	50.10 (6.53, 149.30)		6.58 (3.30, 14.95)	60.70 (8.87, 171.10)
**GFAP**	n=118	n=70	n=48	0.002	n=7	n=41
	212.51 (109.00, 816.76)	174.85 (79.63, 401.27)	469.88 (137.70, 2780.48)		311.21 (159.02, 445.62)	621.39 (136.06, 3009.46)
**UCH-L1**	n=118	n=70	n=48	<0.001	n=7	n=41
	138.95 (40.04, 518.77)	73.39 (25.36, 230.77)	310.40 (110.73, 848.36)		57.50 (19.73, 89.71)	399.78 (171.22, 915.71)

All biomarker concentrations are in pg/ml. GFAP, Glial fibrillary acidic protein; UCH-L1, ubiquitin carboxyl-terminal esterase- L1; NfL, neurofilament light

© 2022 Fink EL et al. *JAMA Network Open*.

B. Day 2

Median (interquartile range)	**Overall N=120**	**Favorable N=70**	**Unfavorable N=50**	**p-value**	**VABS < 70 N=7**	**Dead N=43**
**NFL**	n=104	n=61	n=43	<0.001	n=6	n=37
	75.45 (20.08, 299.66)	31.17 (13.15, 88.67)	287.26 (78.23, 898.47)		147.04 (66.09, 1169.23)	303.00 (126.50, 786.34)
**Tau**	n=104	n=61	n=43	<0.001	n=6	n=37
	15.17 (2.40, 108.41)	5.04 (1.34, 20.23)	99.59 (10.09, 702.00)		24.46 (1.83, 102.81)	114.00 (10.15, 1296.00)
**GFAP**	n=104	n=61	n=43	<0.001	n=6	n=37
	342.01 (139.43, 1912.20)	162.39 (104.46, 361.89)	1515.27 (386.16, 16470.00)		407.60 (283.30, 8109.00)	1575.70 (584.00, 16470.00)
**UCH-L1**	n=104	n=61	n=43	<0.001	n=6	n=37
	94.18 (32.80, 285.75)	51.31 (22.85, 98.31)	319.10 (163.24, 2129.17)		194.52 (27.30, 292.52)	410.87 (172.72, 3163.00)

All biomarker concentrations are in pg/ml. GFAP, Glial fibrillary acidic protein; UCH-L1, ubiquitin carboxyl-terminal esterase- L1; NfL, neurofilament light

© 2022 Fink EL et al. *JAMA Network Open*.

C. Day 3

Median (interquartile range)	**Overall N=120**	**Favorable N=70**	**Unfavorable N=50**	**p-value**	**VABS < 70 N=7**	**Dead N=43**
**NFL**	n=94	n=55	n=39	<0.001	n=6	n=33
	177.60 (35.33, 469.38)	60.63 (23.59, 217.99)	490.88 (136.52, 1512.99)		232.58 (95.99, 1359.43)	528.00 (199.87, 1512.99)
**Tau**	n=94	n=55	n=39	<0.001	n=6	n=33
	15.44 (2.63, 103.01)	6.92 (1.78, 29.16)	97.65 (4.33, 964.00)		103.07 (2.12, 178.22)	93.30 (8.32, 964.00)
**GFAP**	n=93	n=55	n=38	<0.001	n=6	n=32
	334.30 (150.55, 5205.00)	166.21 (98.36, 404.55)	2736.71 (368.75, 12812.05)		1599.75 (347.03, 7303.49)	3543.37 (382.99, 17253.52)
**UCH-L1**	n=93	n=55	n=38	<0.001	n=5	n=33
	117.72 (32.75, 344.54)	49.67 (21.43, 133.10)	357.36 (188.96, 1737.00)		341.60 (16.57, 358.88)	389.02 (192.96, 1737.00)

All biomarker concentrations are in pg/ml. GFAP, Glial fibrillary acidic protein; UCH-L1, ubiquitin carboxyl-terminal esterase- L1; NfL, neurofilament light

P-value, Kruskal-Wallis test, Favorable vs. Unfavorable Outcome

VABS, Vineland Adaptive Behavior Scales

© 2022 Fink EL et al. *JAMA Network Open*.

eTable 3. Biomarker Area Under the Receiver Operating Characteristic Curves (AUROCs), Specificity, Threshold (pg/mL), and Sensitivity on Univariate Biomarker Models, With Set Specificity of 95% for Favorable vs Unfavorable Outcome

A. Day 1

Model	AUROC	Specificity (95%CI)	Threshold level		Sensitivity (95%CI)	Patients, N (%)				
			(pg/mL)	Base 10 logarithm		True Positive	False Negative	False Positive	True Negative	Total
**NFL**	0.731 (0.624, 0.820)	0.957 (0.880, 0.991)	184.00	2.26	0.250 (0.136, 0.396)	12 (10.17)	36 (30.51)	3 (2.54)	67 (56.78)	118
**Tau**	0.704 (0.646, 0.831)	0.957 (0.880, 0.991)	111.75	2.05	0.333 (0.204, 0.484	16 (13.56)	32 (27.12)	3 (2.54)	67 (56.78)	118
**GFAP**	0.670 (0.571, 0.768)	0.957 (0.880, 0.991)	129544.00	5.11	0.043 (0.005, 0.143)	2 (1.69)	46 (38.98)	3 (2.54)	67 (56.78)	118
**UCH-L1**	0.708 (0.614, 0.803)	0.957 (0.878, 0.991)	2319.00	3.37	0.146 (0.061, 0.278)	7 (5.98)	41 (35.04)	3 (2.56)	66 (56.41)	117

Biomarkers on base-10 logarithm scale.

© 2022 Fink EL et al. *JAMA Network Open*.

B. Day 2

Model	AUROC	Specificity (95%CI)	Threshold level		Sensitivity (95%CI)	Patients, N (%)				
			(pg/mL)	Base 10 logarithm		True Positive	False Negative	False Positive	True Negative	Total
**NFL**	0.857 (0.788, 0.926)	0.951 (0.863, 0.990)	389.01	2.59	0.442 (0.291, 0.601)	19 (18.27)	24 (23.08)	3 (2.88)	58 (55.77)	104
**Tau**	0.780 (0.687, 0.874)	0.951 (0.863, 0.990)	240.15	2.38	0.395 (0.259, 0.556)	17 (16.35)	26 (25.00)	3 (2.88)	58 (55.77)	104
**GFAP**	0.795 (0.707, 0.883)	0.951 (0.863, 0.990)	92690.00	4.97	0.12 (0.04, 0.25)	5 (4.81)	38 (36.54)	3 (2.88)	58 (55.77)	104
**UCH-L1**	0.860 (0.785, 0.935)	0.948 (0.856, 0.989)	276.15	2.44	0.581 (0.421, 0.730)	25 (24.75)	18 (17.82)	3 (2.97)	55 (54.46)	101

Biomarkers on base-10 logarithm scale.

© 2022 Fink EL et al. *JAMA Network Open*.

C. Day 3

Model	AUROC	Specificity (95%CI)	Threshold level		Sensitivity (95%CI)	Patients, N (%)				
			(pg/mL)	Base 10 logarithm		True Positive	False Negative	False Positive	True Negative	Total
**NFL**	0.824 (0.742, 0.907)	0.946 (0.849, 0.989)	490.88	2.69	0.513 (0.348, 0.676)	20 (21.28)	19 (20.21)	3 (3.19)	52 (55.32)	94
**Tau**	0.752 (0.647, 0.857)	0.946 (0.849, 0.989)	108.48	2.04	0.487 (0.324, 0.652)	19 (20.21)	20 (21.28)	3 (3.19)	52 (55.32)	94
**GFAP**	0.807 (0.716, 0.897)	0.946 (0.849, 0.989)	40040.00	4.60	0.211 (0.096, 0.373)	8 (8.60)	30 (32.26)	3 (3.23)	52 (55.91)	93
**UCH-L1**	0.837 (0.747, 0.926)	0.962 (0.870, 0.995)	341.60	2.53	0.605 (0.434, 0.760)	23 (25.27)	15 (16.48)	2 (2.20)	51 (56.04)	91

Biomarkers on base-10 logarithm scale.

© 2022 Fink EL et al. *JAMA Network Open*.

eTable 4. Univariate Logistic Regression Models for Favorable or Unfavorable Outcome at 1 Year by Blood-Based Brain Injury Biomarker on Post–Cardiac Arrest Days 1, 2, and 3

A. Neurofilament light

	Day 1		Day 2		Day 3	
	**Odds Ratio**	**95% Wald CI**	**Odds Ratio**	**95% Wald CI**	**Odds Ratio**	**95% Wald CI**
Neurofilament light, log pg/ml	4.23	2.12, 8.45	11.55	4.52, 29.54	7.76	3.22, 18.69
Age, years	0.98	0.92, 1.04	0.98	0.92, 1.04	0.98	0.92, 1.04
Male vs Female sex	0.92	0.44, 1.93	0.92	0.44, 1.93	0.92	0.44, 1.93
Non-White vs White Race	0.82	0.38, 1.79	0.82	0.38, 1.79	0.82	0.38, 1.79
Cardiac vs Asphyxia etiology	0.89	0.38, 2.07	0.89	0.38, 2.07	0.89	0.38, 2.07
In-hospital vs Out of hospital event	0.24	0.11, 0.52	0.24	0.11, 0.52	0.24	0.11, 0.52
Witnessed	0.09	0.03, 0.26	0.09	0.03, 0.26	0.09	0.03, 0.26
Bystander resuscitation	3.38	1.54, 7.41	3.38	1.54, 7.41	3.38	1.54, 7.41
Extracorporeal membrane oxygenation	0.72	0.30, 1.73	0.72	0.30, 1.73	0.72	0.30, 1.73
Pediatric Index of Mortality score	2.10	1.53, 2.89	2.10	1.53, 2.89	2.10	1.53, 2.89
TTM used for prevention of fever	2.47	1.07, 5.68	2.47	1.07, 5.68	2.47	1.07, 5.68
TTM used for therapeutic hypothermia	0.31	0.08, 1.16	0.31	0.08, 1.16	0.31	0.08, 1.16

TTM, targeted temperature management; CI, confidence interval; NA, not applicable

© 2022 Fink EL et al. *JAMA Network Open*.

B. Ubiquitin carboxyl-terminal esterase- L1

	Day 1		Day 2		Day 3	
	**Odds Ratio**	**95% Wald CI**	**Odds Ratio**	**95% Wald CI**	**Odds Ratio**	**95% Wald CI**
Ubiquitin carboxyl-terminal esterase- L1, log pg/ml	2.85	1.61, 5.05	16.32	5.13, 51.92	7.14	2.90, 17.60
Age, years	0.98	0.92, 1.04	0.98	0.92, 1.04	0.98	0.92, 1.04
Male vs Female sex	0.92	0.44, 1.93	0.92	0.44, 1.93	0.92	0.44, 1.93
Non-White vs White Race	0.82	0.38, 1.79	0.82	0.38, 1.79	0.82	0.38, 1.79
Cardiac vs Asphyxia etiology	0.89	0.38, 2.07	0.89	0.38, 2.07	0.89	0.38, 2.07
In-hospital vs Out of hospital event	0.24	0.11, 0.52	0.24	0.11, 0.52	0.24	0.11, 0.52
Witnessed	0.09	0.03, 0.26	0.09	0.03, 0.26	0.09	0.03, 0.26
Bystander resuscitation	3.38	1.54, 7.41	3.38	1.54, 7.41	3.38	1.54, 7.41
Extracorporeal membrane oxygenation	0.72	0.30, 1.73	0.72	0.30, 1.73	0.72	0.30, 1.73
Pediatric Index of Mortality score	2.10	1.53, 2.89	2.10	1.53, 2.89	2.10	1.53, 2.89
TTM used for prevention of fever	2.47	1.07, 5.68	2.47	1.07, 5.68	2.47	1.07, 5.68
TTM used for therapeutic hypothermia	0.31	0.08, 1.16	0.31	0.08, 1.16	0.31	0.08, 1.16

TTM, targeted temperature management; CI, confidence interval; NA, not applicable

© 2022 Fink EL et al. *JAMA Network Open*.

C. Glial fibrillary acidic protein

	Day 1		Day 2		Day 3	
	**Odds Ratio**	**95% Wald CI**	**Odds Ratio**	**95% Wald CI**	**Odds Ratio**	**95% Wald CI**
Glial fibrillary acidic protein, log pg/ml	1.69	1.11, 2.57	2.57	1.60, 4.14	2.74	1.66, 4.50
Age, years	0.98	0.92, 1.04	0.98	0.92, 1.04	0.98	0.92, 1.04
Male vs Female sex	0.92	0.44, 1.93	0.92	0.44, 1.93	0.92	0.44, 1.93
Non-White vs White Race	0.82	0.38, 1.79	0.82	0.38, 1.79	0.82	0.38, 1.79
Cardiac vs Asphyxia etiology	0.89	0.38, 2.07	0.89	0.38, 2.07	0.89	0.38, 2.07
In-hospital vs Out of hospital event	0.24	0.11, 0.52	0.24	0.11, 0.52	0.24	0.11, 0.52
Witnessed	0.09	0.03, 0.26	0.09	0.03, 0.26	0.09	0.03, 0.26
Bystander resuscitation	3.38	1.54, 7.41	3.38	1.54, 7.41	3.38	1.54, 7.41
Extracorporeal membrane oxygenation	0.72	0.30, 1.73	0.72	0.30, 1.73	0.72	0.30, 1.73
Pediatric Index of Mortality score	2.10	1.53, 2.89	2.10	1.53, 2.89	2.10	1.53, 2.89
TTM used for prevention of fever	2.47	1.07, 5.68	2.47	1.07, 5.68	2.47	1.07, 5.68
TTM used for therapeutic hypothermia	0.31	0.08, 1.16	0.31	0.08, 1.16	0.31	0.08, 1.16

TTM, targeted temperature management; CI, confidence interval; NA, not applicable

© 2022 Fink EL et al. *JAMA Network Open*.

D. Tau

	Day 1		Day 2		Day 3	
	**Odds Ratio**	**95% Wald CI**	**Odds Ratio**	**95% Wald CI**	**Odds Ratio**	**95% Wald CI**
Tau, log pg/ml	2.97	1.78, 4.96	3.03	1.88, 4.90	2.59	1.63, 4.12
Age, years	0.98	0.92, 1.04	0.98	0.92, 1.04	0.98	0.92, 1.04
Male vs Female sex	0.92	0.44, 1.93	0.92	0.44, 1.93	0.92	0.44, 1.93
Non-White vs White Race	0.82	0.38, 1.79	0.82	0.38, 1.79	0.82	0.38, 1.79
Cardiac vs Asphyxia etiology	0.89	0.38, 2.0)	0.89	0.38, 2.07	0.89	0.38, 2.07
In-hospital vs Out of hospital event	0.24	0.11, 0.52	0.24	0.11, 0.52	0.24	0.11, 0.52
Witnessed	0.09	0.03, 0.26	0.09	0.03, 0.26	0.09	0.03, 0.26
Bystander resuscitation	3.38	1.54, 7.41	3.38	1.54, 7.41	3.38	1.54, 7.41
Extracorporeal membrane oxygenation	0.72	0.30, 1.73	0.72	0.30, 1.73	0.72	0.30, 1.73
Pediatric Index of Mortality score	2.10	1.53, 2.89	2.10	1.53, 2.89	2.10	1.53, 2.89
TTM used for prevention of fever	2.47	1.07, 5.68	2.47	1.07, 5.68	2.47	1.07, 5.68
TTM used for therapeutic hypothermia	0.31	0.08, 1.16	0.31	0.08, 1.16	0.31	0.08, 1.16

TTM, targeted temperature management; CI, confidence interval; NA, not applicable

© 2022 Fink EL et al. *JAMA Network Open*.

eTable 5. Multivariate Area Under the Receiver Operating Characteristic Curves (AUROCs) With and Without Individual Biomarkers on Days 1, 2, and 3

**Day post-cardiac arrest, biomarker**	**AUROC with biomarker** [a]	**AUROC without biomarker** [a]	**p-value** [b]
Day 1, NfL	0.912	0.886	0.162
Day 1, UCH-L1	0.894	0.886	0.496
Day 1, GFAP	0.893	0.886	0.338
Day 1, Tau	0.895	0.886	0.579
Day 2, NfL	0.932	0.871	0.017
Day 2, UCH-L1	0.918	0.875	0.055
Day 2, GFAP	0.890	0.871	0.250
Day 2, Tau	0.895	0.871	0.248
Day 3, NfL	0.921	0.870	0.034
Day 3, UCH-L1	0.904	0.867	0.165
Day 3, GFAP	0.899	0.865	0.069
Day 3, Tau	0.881	0.870	0.534

NfL, Neurofilament light; UCH-L1, Ubiquitin carboxyl-terminal esterase- L1; GFAP, Glial fibrillary acidic protein

[a] p<.2, after adjusting for age, sex, race, etiology, location, witnessed, bystander status, ECMO, severity of illness (PIM3), TTM for fever or hypothermia

[b] Chi-square analysis of AUROC between models with and without biomarker

© 2022 Fink EL et al. *JAMA Network Open*.

eTable 6. Comparison of Patient and Cardiac Arrest Characteristics Among Children With and Without Data Available on 1 Year Outcomes

N (%) or Median (interquartile range)	**One year outcome obtained N=120**	**One year outcome not obtained N=43**	**p-value**
**Age, y**	1.0 (0.0, 8.5)	3.0 (1.0, 10.0)	0.039
**Sex**			0.590
Female	49 (40.8)	20 (46.5)	
Male	71 (59.2)	33 (53.5)	
**Race**	n=120	n=43	<.001
Asian	5 (4.2)	0 (0)	
Black	19 (15.8)	17 (39.5)	
White	81 (67.5)	23 (53.5)	
Unknown	15 (12.5)	3 (7.0)	
**Hispanic Ethnicity**	11 (10.0), n=110	3 (7.1), n=42	0.759
**Pre-existing conditions** [1]	77 (68.1), n=113	26 (60.5), n=43	0.450
Cardiovascular	45 (37.8), n=119	6 (14.0), n=43	0.004
Pulmonary disease	25 (20.8), n=120	10 (23.3), n=43	0.829
Premature birth	24 (21.8), n=110	6 (15.4), n=39	0.489
Congenital syndrome	14 (11.8), n=119	4 (9.3), n=43	0.783
Neurologic	11 (9.2), n=120	4 (9.3), n=43	1.000
Oncologic syndrome	3 (2.5), n=120	1 (2.3), n=43	1.000
Organ or cellular transplant	3 (2.5), n=119	1 (2.3), n=43	1.000
**Primary etiology**	n=107	n=41	0.091
Asphyxia	74 (69.2)	34 (82.9)	
Cardiac	33 (30.8)	7 (17.1)	
**Location, out of hospital**	60 (50.0)	30 (69.8)	0.032
**Duration of cardiopulmonary resuscitation, minutes**	7.00 (3.00, 20.00), n=99	7.00 (2.00, 17.00), n=33	0.596
**Total number of epinephrine doses**	1.0 (0.0, 3.0), n=100	0.0 (0.0, 2.0), n=37	0.031
**Defibrillated**	18 (17.8), n=101	7 (18.4), n=38	1.000
**First monitored rhythm**	n=96	n=28	0.030
Sinus bradycardia	33 (34.4)	11 (39.3)	
Pulseless electrical activity	23 (24.0)	6 (21.4)	

© 2022 Fink EL et al. *JAMA Network Open*.

Asystole	19 (19.8)	1 (3.6)	
Ventricular tachycardia or fibrillation	15 (15.6)	3 (10.7)	
Sinus tachycardia/Junctional ectopic tachycardia	4 (4.2)	3 (10.7)	
Normal sinus rhythm	2 (2.1)	4 (14.3)	
**Witnessed**	92 (76.7)	29 (67.4)	0.309
**Bystander resuscitation**	42 (35.0)	23 (53.5)	0.045
**Hospital length of stay, d**	18.0 (6.5, 35.5), n=120	10.0 (6.0, 20.0), n=42	0.033
**Intensive care unit length of stay, d**	12.0 (5.0, 25.0), n=117	8.0 (2.5, 11.0), n=40	0.002
**Disposition at hospital discharge**	n=120	n=42	<.001
Home	55 (45.8)	33 (78.6)	
Died	41 (34.2)	0 (0)	
Inpatient rehabilitation	17 (14.2)	6 (14.3)	
Transfer to other hospital	2 (1.7)	3 (7.1)	
Long-term care facility	5 (4.2)	0 (0)	
**Days from cardiac arrest event to death**	10 (3, 25), n=43	NA	
**Cause of death**	n=43	n=0	
Multiple Organ Failure	13 (30.2)	NA	
Brain death	11 (25.6)	NA	
Neurologic injury	11 (25.6)	NA	
Cardiovascular	8 (18.6)	NA	

[1] Children may have more than one pre-existing condition

p-value are based on Chi-square test for categorical variables, Kruskal-Wallis test for median

© 2022 Fink EL et al. *JAMA Network Open*.
