# Supplementary material for: Association of Blood-Based Brain Injury Biomarker Concentrations With Outcomes After Pediatric Cardiac Arrest
Source: JAMA Netw Open. 2022 Sep 8;5(9):e2230518. doi: 10.1001/jamanetworkopen.2022.30518 (PMC9459665; doi:10.1001/jamanetworkopen.2022.30518)
Supplement: Supplement 2. — Nonauthor Collaborators. Personalizing Outcomes After Child Cardiac Arrest (POCCA) Investigators [file jamanetwopen-e2230518-s002.pdf]

Supplemental Online Content: Nonauthor Collaborators
*First name, last name, and suffix (if applicable) are required and will appear in PubMed.

*Group Name(s): Personalizing Outcomes After Child Cardiac Arrest (POCCA) Investigators							
***First Name and Middle Initial(s)**	***Last Name**	***Suffix (eg, Jr, III)**	Academic Degrees	Institution	Location (city, state/province, country)	Role or Contribution, eg, chair, principal investigator	Group (if more than 1 Group listed in the byline) and/or Subgroup (eg, Steering Committee)
David	Maloney		BS	UPMC Children's Hospital of Pitts[illegible]	Pittsburgh, PA, USA	Research Coordinator	
Pamela	Rubin		RN	UPMC Children's Hospital of Pitts[illegible]	Pittsburgh, PA, USA	Research Coordinator	
Beena	Desai		BS, CCRC	Children's Healthcare of Atlanta	Atlanta, GA, USA	Research Coordinator	
Maureen G.	Richardson		BSN, RN, [illegible]	Children's Healthcare of Atlanta	Atlanta, GA, USA	Research Coordinator	
Cynthia	Bates		CCRP	Children's Healthcare of Atlanta	Atlanta, GA, USA	Research Coordinator	
Darshana	Parikh			Children's Hospital of Philadelphi[illegible]	Philadelphia, PA, USA	Research Coordinator	
Janice	Prodell			Children's Hospital of Philadelphi[illegible]	Philadelphia, PA, USA	Research Coordinator	
Maddie	Winters			Children's Hospital of Philadelphi[illegible]	Philadelphia, PA, USA	Research Coordinator	
Katherine	Smith		MPH, BSN	Children's Hospital of Philadelphi[illegible]	Philadelphia, PA, USA	Research Coordinator	
Jeni	Kwok		JD	Children's Hospital of Los Angeles	Los Angeles, CA, USA	Research Coordinator	
Adriana	Cabrales		BA	Children's Hospital of Los Angeles	Los Angeles, CA, USA	Research Coordinator	
Ronke	Adewale			Johns Hopkins Children's Center	Baltimore, MD, USA	Research Coordinator	
Pam	Melvin			Johns Hopkins Children's Center	Baltimore, MD, USA	Research Coordinator	
Sadaf	Shad			Children's Hospital of Wisconsin	Milwaukee, WI, USA	Research Coordinator	
Katherine	Siegel			Children's Hospital of Wisconsin	Milwaukee, WI, USA	Research Coordinator	

Supplemental Online Content: Nonauthor Collaborators
*First name, last name, and suffix (if applicable) are required and will appear in PubMed.

***First Name and Middle Initial(s)**	***Last Name**	***Suffix (eg, Jr, III)**	Academic Degrees	Institution	Location (city, state/province, country)	Role or Contribution, eg, chair, principal investigator	Group (if more than 1 Group listed in the byline) and/or Subgroup (eg, Steering Committee)
Katherine	Murkowski			Children's Hospital of Wisconsin	Milwaukee, WI, USA	Research Coordinator	
Mary	Kasch			Children's Hospital of Wisconsin	Milwaukee, WI, USA	Research Coordinator	
Josey	Hensley		RN	Nationwide Children's Hospital	Columbus, OH, USA	Research Coordinator	
Lisa	Steele		RN, BSN	Nationwide Children's Hospital	Columbus, OH, USA	Research Coordinator	
Danielle	Brown			Phoenix Children's Hospital	Phoenix, AZ, USA	Research Coordinator	
Brian	Burrows			Phoenix Children's Hospital	Phoenix, AZ, USA	Research Coordinator	
Lauren	Hlivka			Phoenix Children's Hospital	Phoenix, AZ, USA	Research Coordinator	
Deana	Rich			Seattle Children's Hospital	Seattle, WA, USA	Research Coordinator	
Amila	Tutundzic			St. Louis Children's Hospital	St. Louis, MO, USA	Research Coordinator	
Tina	Day			St. Louis Children's Hospital	St. Louis, MO, USA	Research Coordinator	
Lori	Barganier			St. Louis Children's Hospital	St. Louis, MO, USA	Research Coordinator	
Ashley	Wolfe			Children's National Hospital	Washington, DC, USA	Research Coordinator	
Mackenzie	Little			Children's National Hospital	Washington, DC, USA	Research Coordinator	
Elyse	Tomanio			Children's National Hospital	Washington, DC, USA	Research Coordinator	
Neha	Patel			Children's National Hospital	Washington, DC, USA	Research Coordinator	
Diane	Hession			Children's National Hospital	Washington, DC, USA	Research Coordinator	

Supplemental Online Content: Nonauthor Collaborators
*First name, last name, and suffix (if applicable) are required and will appear in PubMed.

***First Name and Middle Initial(s)**	***Last Name**	***Suffix (eg, Jr, III)**	Academic Degrees	Institution	Location (city, state/province, country)	Role or Contribution, eg, chair, principal investigator	Group (if more than 1 Group listed in the byline) and/or Subgroup (eg, Steering Committee)
Yamila	Sierra		MPH, CCR	Children's Hospital of Colorado	Denver, CO, USA	Research Coordinator	
Rhonda	Jones			Cincinnati Children's Hospital Me	Cincinnati, OH, USA	Research Coordinator	
Laura	Benken			Cincinnati Children's Hospital Me	Cincinnati, OH, USA	Research Coordinator	
Jonathan	Elmer		MD, MS		Pittsburgh, PA, USA	Collaborator	
Subramanian	Subramanian		MD		Pittsburgh, PA, USA	Pediatric Neuroradiologist	
Srikala	Narayanan		MD		Pittsburgh, PA, USA	Pediatric Neuroradiologist	
Nicole	Toney		MPH		Pittsburgh, PA, USA	Research Coordinator	
Julia	Wallace				Pittsburgh, PA, USA	Research Coordinator	
Tami	Robinson				Pittsburgh, PA, USA	Data Manager	
Andrew	Frank				San Francisco, CA, USA	Imaging storage expert	
Stefan	Bluml		PhD		Los Angeles, CA, USA	MR Spectroscopist	
Jessica	Wisnowski		PhD		Los Angeles, CA, USA	MR Spectroscopist	
Keri	Feldman				Pittsburgh, PA, USA	Laboratory technician	
Avinash	Vemulapalli				Pittsburgh, PA, USA	Data Manager	
Linda	Ryan				Pittsburgh, PA, USA	Grants administrator	
Scott	Szypulski		MBA		Pittsburgh, PA, USA	Grants administrator	
Christopher	Keys				Pittsburgh, PA, USA	Grants administrator	
